# Biodiversity assessments: Origin matters

**DOI:** 10.1371/journal.pbio.2006686

**Published:** 2018-11-13

**Authors:** Aníbal Pauchard, Laura A. Meyerson, Sven Bacher, Tim M. Blackburn, Giuseppe Brundu, Marc W. Cadotte, Franck Courchamp, Franz Essl, Piero Genovesi, Sylvia Haider, Nick D. Holmes, Philip E. Hulme, Jonathan M. Jeschke, Julie L. Lockwood, Ana Novoa, Martin A. Nuñez, Duane A. Peltzer, Petr Pyšek, David M. Richardson, Daniel Simberloff, Kevin Smith, Brian W. van Wilgen, Montserrat Vilà, John R. U. Wilson, Marten Winter, Rafael D. Zenni

**Affiliations:** 1 Facultad de Ciencias Forestales, Universidad de Concepción, Concepción, Chile; 2 Institute of Ecology and Biodiversity, Santiago, Chile; 3 University of Rhode Island, Natural Resources Science, Kingston, Rhode Island, United States of America; 4 Department of Biology, University of Fribourg, Fribourg, Switzerland; 5 Centre for Biodiversity and Environment Research, Department of Genetics, Evolution, and Environment, University College London, London, United Kingdom; 6 Institute of Zoology, Zoological Society of London, Regent’s Park, London, United Kingdom; 7 University of Sassari, Department of Agriculture, Sassari, Italy; 8 University of Toronto, Scarborough, Toronto, Canada; 9 Ecologie, Systématique, et Evolution, Univ Paris-Sud, CNRS, AgroParisTech, Université Paris-Saclay, Paris, France; 10 Division of Conservation Biology, Vegetation and Landscape Ecology, University Vienna, Vienna, Austria; 11 ISPRA, Institute for Environmental Protection and Research and Chair IUCN SSC Invasive Species Specialist Group, Rome, Italy; 12 Martin Luther University Halle-Wittenberg, Institute of Biology/Geobotany and Botanical Garden, Halle, Germany; 13 German Centre for Integrative Biodiversity Research (iDiv) Halle-Jena-Leipzig, Leipzig, Germany; 14 Island Conservation, Santa Cruz, California, United States of America; 15 Bio-Protection Research Centre, Lincoln University, Canterbury, New Zealand; 16 Leibniz-Institute of Freshwater Ecology and Inland Fisheries, Berlin, Germany; 17 Institute of Biology, Freie Universität Berlin, Berlin, Germany; 18 Ecology, Evolution, and Natural Resources, Rutgers University, New Brunswick, New Jersey, United States of America; 19 Department of Invasion Ecology, Institute of Botany, Czech Academy of Sciences, Prague, Czech Republic; 20 Grupo de Ecologıa de Invasiones, INIBIOMA, CONICET Universidad Nacional del Comahue, Argentina; 21 Manaaki Whenua Landcare Research, Lincoln, New Zealand; 22 Department of Ecology, Faculty of Science, Charles University, Prague, Czech Republic; 23 Centre for Invasion Biology, Department of Botany and Zoology, Stellenbosch University, Matieland, South Africa; 24 Department of Ecology and Evolutionary Biology, University of Tennessee, Knoxville, Tennessee, United States of America; 25 International Union for Conservation of Nature, Cambridge, United Kingdom; 26 Estación Biológica de Doñana (EBD-CSIC), Isla de la Cartuja, Sevilla, Spain; 27 South African National Biodiversity Institute, South Africa, Stellenbosch, South Africa; 28 Department of Biology, University of Lavras, Lavras, Minas Gerais, Brazil

Recent global efforts in biodiversity accounting, such as those undertaken through the Convention on Biological Diversity (CBD) and Intergovernmental Science-Policy Platform on Biodiversity and Ecosystem Services (IPBES), are vital if we are to track conservation progress, ensure that we can address the challenges of global change, and develop powerful and scientifically sound indicators. Schlaepfer [1] proposes that we should work toward inventories of biodiversity that account for native and non-native species regardless of species origin and ecological context. We strongly disagree with the approach of combining counts of native and non-native species because this will reduce our capacity to detect the effects of non-native species on native biodiversity with potentially devastating consequences. Compelling and abundant evidence demonstrates that some non-native species can become invasive and produce major ecosystem disruptions and even native species extinction. Unfortunately, we still cannot be certain which non-native species will be the most detrimental (e.g., [2]). Combining native and non-native species together into a single biodiversity index would not only inflate biodiversity estimates and risk promoting the spread of invasive non-native species but would also ignore the fundamental ecological differences between the two groups. The critical differences that should be considered when assessing biodiversity include the following.

## 1. Evolutionary history

Native species have coevolved with one another and the physical environment, often resulting in intricate coadaptations [3]. Loss of native species can erase unique evolutionary histories. Therefore, non-native species additions do not compensate for phylogenetic losses resulting from extinctions even if they increase overall local species diversity, because many non-native species erode diversity through local and global extinctions [4]. Even if one is willing to offset the current losses of biodiversity with the promise of new biodiversity as non-native species evolve and diverge, millions of years of biological adaptation and evolutionary history would be lost.

## 2. Biotic homogenization

Biogeographic isolation is a fundamental driver of the Earth’s biodiversity. Long-standing physical and biological isolation of continents, islands, and marine realms has produced extant biogeographic patterns, including hotspots of endemism and species richness. This is the natural heritage that biodiversity assessments seek to document and that global conservation policy seeks to maintain. The widespread introduction of non-native species, especially damaging invasive non-native species, tends to homogenize biodiversity so that regions lose their biological distinctiveness at taxonomic, functional, and phylogenetic levels [5].

## 3. Ecosystem functions and services

The species that make up an ecosystem contribute to a multitude of functions and services, and the diversity of trait combinations ensures ecosystem multifunctionality over time and under changing environmental conditions [6]. Many ecosystem services are underappreciated by humans, and some critical ecosystem functions are surely yet to be discovered. While single ecosystem functions might be replaced by non-native species, non-native species can also decrease the value or rate of overall ecosystem service provisioning [2]. Further, our understanding of time lags and temporal dynamics of non-native species’ impacts indicates that non-native species that appear harmless today may not remain so in the future [7]. We lack the ability to predict which currently benign non-native species will be future invaders [8]. What appears as an advantage now (i.e., new species additions) may weaken biosphere sustainability over the longer term.

Designating the origin of species in biodiversity assessments is imperative to understanding the state of global biodiversity. The fact that humans use and benefit from some non-native species does not erase the negative impacts that many other non-native species have on biodiversity, ecosystem services, and human well-being. Biodiversity indices, particularly coarse proxies such as species numbers, should be transparently calculated and carefully interpreted before drawing broad generalizations. In assessments of how much we have modified or destroyed our natural capital, changes in native species dynamics are one of the most relevant indicators. The indices reviewed in Schlaepfer’s paper [1] do not consider non-native species because they were largely designed to monitor the status of native biodiversity. Including non-natives in estimates of total biodiversity without distinguishing their origin will lead to absurd situations in which drivers of biodiversity loss contribute to improvements in the metrics used to evaluate biodiversity conservation.

Moving forward, we agree with Schlaepfer [1] that full inventories of native and non-native biodiversity are needed both globally and for individual countries. For some taxonomic groups, such data are becoming available for most of the world [9]. However, we should not simply tally species in a single column and declare our assessment complete and relevant. We must instead aim for global and local assessments that reflect the complex interplay between species, the physical environment, and human dependence on this complexity [10]. Assessments should not only count all species but also assess their origin, relative abundance, probability of extinction, contribution to biodiversity dynamics, ecosystem services and functions, and role in ecological networks. Both the harmful and beneficial roles of non-native species in providing ecological, human health, and economic benefits deserve special attention [2]. These are ambitious aims given the challenges of producing even simple species lists for many areas, but they are vital if we are to understand the relevance of biodiversity to planetary health.

Through the CBD and the Sustainable Development Goals (SDGs), the world has committed to managing the impacts of priority invasive non-native species and preventing further introductions by 2020 in order to protect biodiversity and address poverty and inequalities. However, progress toward meeting these targets has been insufficient. With the international community beginning to negotiate a new global biodiversity framework to implement after 2020, it is critical that the scientific community provides clear and current objective knowledge of the threats posed by non-native species invasions, the unprecedented rate of new introductions, and their impacts on native biodiversity and human well-being, particularly within emerging economies [11].

Schlaepfer [1] states that “Biodiversity indices will need to encompass all species if they are to remain socially relevant and illustrate the full gamut of what are now called ecosystem services.” Without providing clear guidelines and a concrete proposal for how this could be achieved and supported by rigorous data, this demand lacks value and is potentially dangerous because it opens the door to unscientific and false equivalences, public policy confusion, and societal acceptance of inaction among policymakers and managers. As knowledge and data availability continue to grow, now is the time to develop more comprehensive assessments to quantify the state of global biodiversity and to monitor carefully how native and non-native species are shaping the biosphere. However, in doing so, we must resist the temptation to oversimplify the concept of biodiversity.

## Abbreviations

CBD: Convention on Biological Diversity
IPBES: Intergovernmental Science-Policy Platform on Biodiversity and Ecosystem Services
SDG: Sustainable Development Goal
